# Application of color doppler ultrasound and US shear wave elastography with connective tissue growth factor in the risk assessment of papillary thyroid carcinoma

**DOI:** 10.1186/s12880-024-01354-w

**Published:** 2024-07-12

**Authors:** Xiaoling Leng, Jinhui Liu, Qiao Zou, Changchun Wang, Sen Yang

**Affiliations:** 1 Department of Ultrasonography, The Tenth Affiliated Hospital of Southern Medical University(Dongguan People’s Hospital), Dongguan, Guangdong P.R. China; 2 Department of Ultrasound, The Affiliated Tumor Hospital of Xinjiang Medical University, Urumqi, 830011 P.R. China

**Keywords:** Papillary thyroid carcinoma, Shear wave elastography, Connective tissue growth factor

## Abstract

**Background:**

This study aims to investigate the role of shear wave elastography (SWE) and connective tissue growth factor (CTGF) in the assessment of papillary thyroid carcinoma (PTC) prognosis.

**Methods:**

CTGF expression was detected with immunohistochemistry. Clinical and pathological data were collected. Parameters of conventional ultrasound combined with SWE were also collected. The relationship among CTGF expression, ultrasound indicators, the elastic modulus and the clinicopathological parameters were analyzed.

**Results:**

Univariate analysis showed that patients with high risk of PTC were characterized with male, Uygur ethnicity, increased expression of CTGF, convex lesions, calcified, incomplete capsule, intranodular blood flow, rear echo attenuation, cervical lymph node metastasis, lesions larger than 1 cm, psammoma bodies, advanced clinical stage, increased TSH and high value in the shear modulus (*P* < 0.05). Multivariate analysis demonstrated that the risk factors of high expression of CTGF according to contribution size order were irregular shape, aspect ratio ≥ 1, and increased TSH. The logistic regression model equation was Logit (P) = 1.153 + 1.055 × 1 + 0.926 × 2 + 1.190 × 3 and the Area Under Curve value of the logistic regression was calculated to be 0.850, with a 95% confidence interval of 0.817 to 0.883.

**Conclusion:**

SWE and CTGF are of great value in the risk assessment of PTC. The degree of fibrosis of PTC is closely related to the prognosis. The hardness of PTC lesions and the expression level of CTGF are correlated with the main indexes of conventional ultrasound differentiating benign or malignant nodules. Irregular shape, aspect ratio ≥ 1, and increased TSH are independent factors of CTGF.

## Background

The incidence of papillary thyroid carcinoma (PTC) has been increasing in recent years [1]. The early diagnosis and treatment of PTC has attracted much attention. Similar treatment strategy may result poor outcomes in some patients or excessive treatment in others [2]. Although the ultrasound is considered as the best imaging method to diagnose PTC, its value remains controversial in evaluating extranodal invasion and lymph node metastasis, especially the central lymph node metastasis [3–5].

Shear wave elastography (SWE) is used to calculate the tissue absolute hardness by recording shear wave propagation velocity with the Young’s modulus. The combination of SWE and conventional ultrasound could significantly improve the differential diagnosis in thyroid benign and malignant nodules [6]. SWE could also reflect the pathological structure of tumor based on theory that the lesion tissue hardness is related with the pathological structure [7]. SWE can effectively predict cervical lymph node metastasis in PTC [8].

Connective tissue growth factor (CTGF) plays an important role in malignant tissue fibrosis [9]. The rapid growth of carcinoma is accompanied with interstitial fibrosis and hardening. Previous study found that CTGF promoted cell proliferation and extracellular matrix synthesis and accumulation in the process of fibrosis [10]. The expression of CTGF in thyroid cancer is closely related with tumorigenesis, tumor progression, extracellular matrix formation, metastasis and prognosis [11]. CTGF is an independent prognostic factor for PTC [12].

Previous study mainly focused on the diagnostic and prediction value of elastic modulus of SWE using various parameters in PTC [13]. However, to our knowledge, there are no reports on the combined role of SWE and CTGF in PTC. This study aims to investigate the role of SWE and CTGF in prognosis assessment of PTC.

## Materials and methods

### Study subjects

**Fig. 1:**
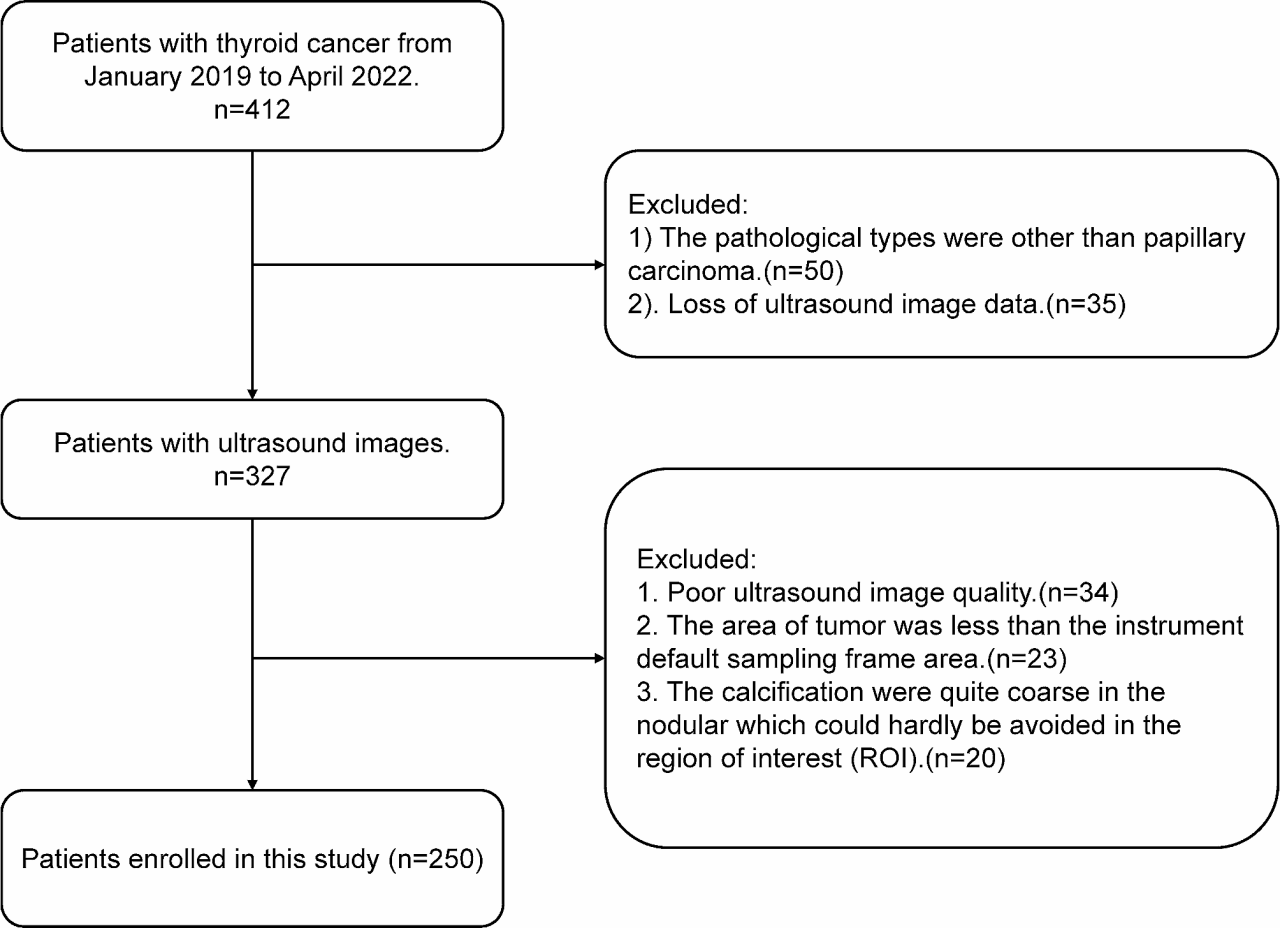
Flowchart of patient enrollment

Following the guidelines of the Thyroid Imaging Reporting and Data System (TI-RADS) released by the American College of Radiology (ACR) in 2017, routine ultrasound and SWE examinations were conducted on all suspected TI-RADS 4 thyroid nodule patients at the Cancer Center of Xinjiang Medical University from 2019 to 2022. Patients were included in this study if they were diagnosed of PTC based on pathological results after surgery dissection. The exclusion criteria were stated as follows: (1) The area of tumor was less than the instrument default sampling frame area; (2) The calcification were quite coarse in the nodular which could hardly be avoided in the region of interest (ROI); (3) The pathological types were other than papillary carcinoma. The flowchart of patient enrollment is shown in Fig. 1. A total of 250 patients were included, in which 172 were women and 78 were men. Their age ranged from 23 to 74 years old with an average of 44.45 ± 0.90 years old. Totally, 306 nodules were detected in this study and their maximum diameter ranged from 5.0 to 73.0 mm with an average of 0.96 ± 0.78 mm. The basic demographic data were collected from all patients including age (≥ 45 /<45 years old), ethnicity (Han/Uygur/others), gender and thyroid stimulating hormone (TSH). The pathological features were collected after operation including tumor size (≥ 1 / <1 cm), lymph node metastasis, clinical stage, psammoma bodies, lesions composition (solid/moderate/soft) and etc. The study protocol was approved by the ethical committee of Xinjiang Tumor Hospital and all the patients signed informed consent.

### SWE examination and image analysis

The patients were in supine position. The ultrasound examinations were performed with SuperSonic Imagine AixPlorer (France) using L4-15 linear probe in a frequency of 4 ~ 15 MHz. The patients underwent a two-dimensional gray-scale and Doppler color flow ultrasound scan. Then a stable image was frozen in SWE mode and the ROI was measured with Q-BOX function to calculate Young’s modulus value, including the minimum (E-min), average (E-mean), maximum (E-max), and standard deviation (E-SD) of Young’s modulus value. The ROI covered the part of the lesions with higher hardness as much as possible or the solid part if the lesion was cystic. The stiffer lesions were displayed in red color, while the softer ones were in blue. Each patient was measured by two experienced ultrasound physicians independently in a double-blinded manner.

All nodules were evaluated and classified according to the following predefined ultrasound features: (1) thyroid background (hashimoto’s thyroiditis / normal), (2) lesions size (≥ 1 /< 1 cm), (3) nodule convex (yes/no), (4) lesion morphology (regular / irregular), (5) margins (clear / blurred), (6) aspect ratio (< 1 / ≥1), (7) internal echoes (homogeneous / heterogeneous), (8) calcification (yes/no), (9) liquefaction necrosis (yes/no), (10) rear-echo attenuation (yes/no), 11) thyroid envelope (intact / incomplete), 12) blood flow (perinodular /intranodular blood flow), and 13) thyroid nodule status (hot/cold thyroid nodules).

### Immunohistochemistry

The expression of CTGF was detected by immunohistochemistry according to routine procedure. Cells with brown particles in the cytoplasma were defined as CTGF positive cells. Ten high power fields (× 400) of each section were selected randomly and positive cells were calculated. The results were defined according to the number of positive cells as follows: (-) there were no positive cells; (+) percentages of positive cells were < 30%; (++) percentages of positive cells were 30 ~ 50% and (+++) percentage of positive cells was > 50%.

### Statistical analysis

The data were analyzed using SPSS 19.0 software. The categorical variables were analyzed with χ2 test. Continuous data were compared with Student’s *t* test or ANOVA. Pearson correlation was used for correlation analysis. Evaluating repeatability by measuring intra-class correlation coefficients (ICCs) suitable for continuous variables: The consistency at low levels approaches 0, while at high levels, it tends toward 1 [14]. ICC values of ≥ 0.9 indicate excellent agreement, 0.70–0.89 indicate good agreement, 0.50–0.69 indicate moderate agreement, 0.30–0.49 indicate fair agreement, and ≤ 0.29 indicate poor agreement [15]. Logistic regression analysis was performed. Variables significantly related to the thyroid lesion hardness in univariate analysis were included in multivariate logistic regression model. The regression model was obtained. The Wald *X*^2^ test was used to estimate the regression parameters, and the likelihood ratio test was used to assess the fitness of the model. *P* < 0.05 was considered statistically significant.

## Results

### Clinicopathological indicators, ultrasound indicators and SWE indicators related to PTC prognosis

According to the relapse risk of differentiated thyroid cancer [16], the patients were stratified into low-risk, medium risk and high-risk group. The clinicopathological, ultrasound and SWE indicators among different groups were compared (Table 1). We found that the factors of male gender, Uighur ethnicity, high expression of CTGF, convex lesions, calcification, incomplete capsule, intranodular blood flow, rear echo attenuation, cervical lymph node metastasis, lesions greater than 1 cm, psammoma bodies, advanced clinical stage, elevated TSH and increased SWE modulus value (E-mean, E-min, E-max and E-SD) were associated with higher risk of PTC (*P* < 0.05). However, prognosis was insignificantly related with factors including age, thyroid gland background, lesion morphology, margin, aspect ratio, liquefaction necrosis, internal echo, halo, thyroid nodule status, pathological texture (*P* > 0.05). Thus, the factors of gender, ethnicity, TSH, CTGF, SWE modulus value, lesion size, calcification, and clinical staging are related with prognosis of PTC. The ICC values for all indices of ultrasound elasticity modulus fell between 0.70 and 0.89, indicating good intra-observer agreement (Table 2).

**Table 1:** The comparison of clinicopathological, ultrasonographic and elastic shear wave characteristics of PTC patients with stratified risk

Characteristics		Risk stratification			X^2^	*P*
		Low risk	Medium risk	High risk		
Gender	Male	16	47	15	23.695	0.001
	Female	86	100	41		
Ethnicity	Han	69	112	43	309.801	< 0.001
	Uighur	19	19	10		
	Others	14	16	5		
CTGF	-	23	15	5	37.065	< 0.001
	+	25	18	2		
	++	35	45	19		
	+++	19	69	31		
BRAF V600E	-	20	26	2	8.022	0.018
	+	82	121	55		
Nodule convex	Yes	1	4	11	31.964	< 0.001
	No	102	142	46		
Calcification	Yes	84	114	54	6.247	0.044
	No	18	32	4		
Capsule	Intact capsule	102	144	36	86.42	< 0.001
	Incomplete capsule	1	2	21		
Blood flow	Perinodular blood flow	65	73	23	9.004	0.011
	Intranodular blood flow	37	74	34		
Rear echo attenuation	Yes	78	122	55	8.777	0.012
	No	24	24	3		
Thyroid nodule status	Hot	27	50	15	2.098	0.350
	Cold	75	97	42		
Cervical lymph nodes metastasis	Yes	5	11	9	7.147	0.028
	No	98	135	48		
Lesion size	< 1 cm	87	95	15	55.472	< 0.001
	≥ 1 cm	15	52	42		
Psammoma bodies	Yes	33	63	34	12.454	0.006
	No	69	84	23		
Clinical stage	Stage 1	94	100	32	184.684	< 0.001
	> Stage 1 (advanced stage)	7	47	25		
TSH	Reduced	9	12	2	157.958	< 0.001
	Normal	71	90	40		
	Increased	21	45	16		
E-mean	> average value	26	86	39	36.452	< 0.001
	≤average value	76	61	18		
E-min	> average value	32	83	35	19.467	< 0.001
	≤average value	70	64	22		
E-max	> average value	28	81	40	31.369	< 0.001
	≤average value	74	66	17		
E-SD	> average value	34	79	39	19.881	< 0.001
	≤average value	68	68	18		

Note: CTGF, connective tissue growth factor; TSH, thyroid stimulating hormone; E-mean/E-min/E-max/E-SD, the mean/minimum/max/ standard deviation value of SWE Young’s modulus value

**Table 2:** Elastic modulus value of PTC with different CTGF expression

Elastic modulus value	*n*	Mean ± SD		ICC	*P*
		Observation 1	Observation 2		
E-mean	68	31.733 ± 20.448	39.157 ± 24.281	0.735	< 0.001^**^
E-min	68	16.830 ± 12.297	20.887 ± 14.365	0.772	< 0.001^**^
E-max	68	45.706 ± 37.281	58.324 ± 42.470	0.772	< 0.001^**^
E-SD	68	6.792 ± 6.991	8.484 ± 9.205	0.839	< 0.001^**^

Note: *p < 0.05, **p < 0.01. E-mean/E-min/E-max/E-SD, the mean/minimum/max/ standard deviation value of SWE Young’s modulus value

### The relationship of PTC elastic modulus with ultrasound and clinicopathological indicators

**Fig. 2:**
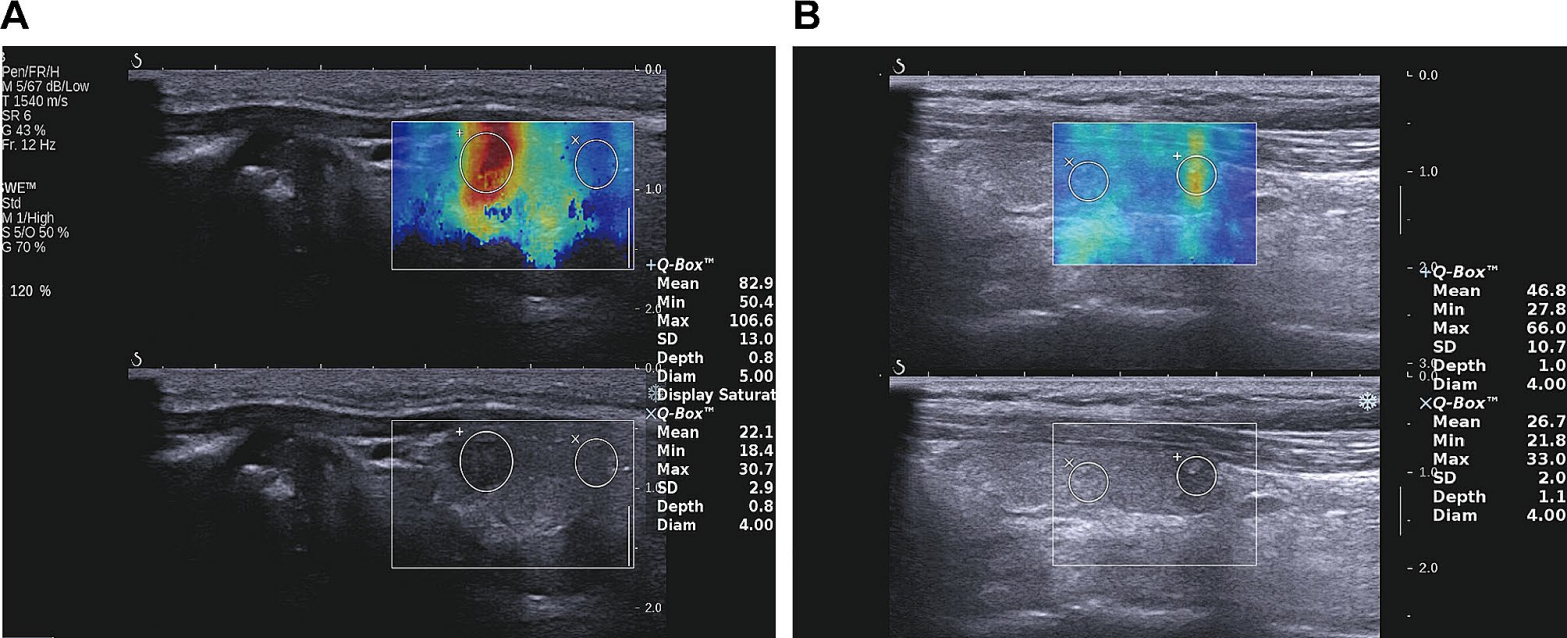
The representative SWE images. The SWE measurement was performed on the lesion. The Q-box was placed in the lesion and normal tissue next to the lesion. **(A)** PTC lesion with high elastic modulus. It was in the lower right lobe of the thyroid gland, with 6 * 4 mm in size. It had convex and irregular shapes, with unclear borders, an aspect ratio greater than 1, and an incomplete thyroid capsule. The diameter of the Q-box in the lesion was 4 mm. The elastic modulus were: E-mean: 56.6 kPa, E-min: 35.5 kPa, E-max: 73.6 kPa, and E-SD: 9.9 kPa. The diameter of the Q-box in the normal tissue was 4.5 mm, with E-mean: 28.3 kPa, E-min: 20.8 kPa, E-max: 36.5 kPa, and E- SD: 4.5 kPa. **(B)** PTC lesion with low elastic modulus. It was middle right lobe of the thyroid, with size of 6 * 4 mm. The lesion was not convex with regular shape. The border was less clear. The aspect ratio was less than 1, and the thyroid capsule was intact. The diameter of the Q-box in the lesion was 4.0 mm. The elasticity values were: E-mean: 40.7 kPa, E-min: 31.9 kPa, E-max: 48.6 kPa, and E-SD: 3.5 kPa. The diameter of the Q-box in normal tissue was 4.0 mm, with E-mean: 14.3.3 kPa, E-min: 10.8 kPa, E-max: 319.3 kPa, and E-SD: 1.9 kPa. The SWE imaging of low Risk PTC. The normal tissue was selected as control. All parameters of nodular stiffness were similar to normal tissue

**Fig. 3:**
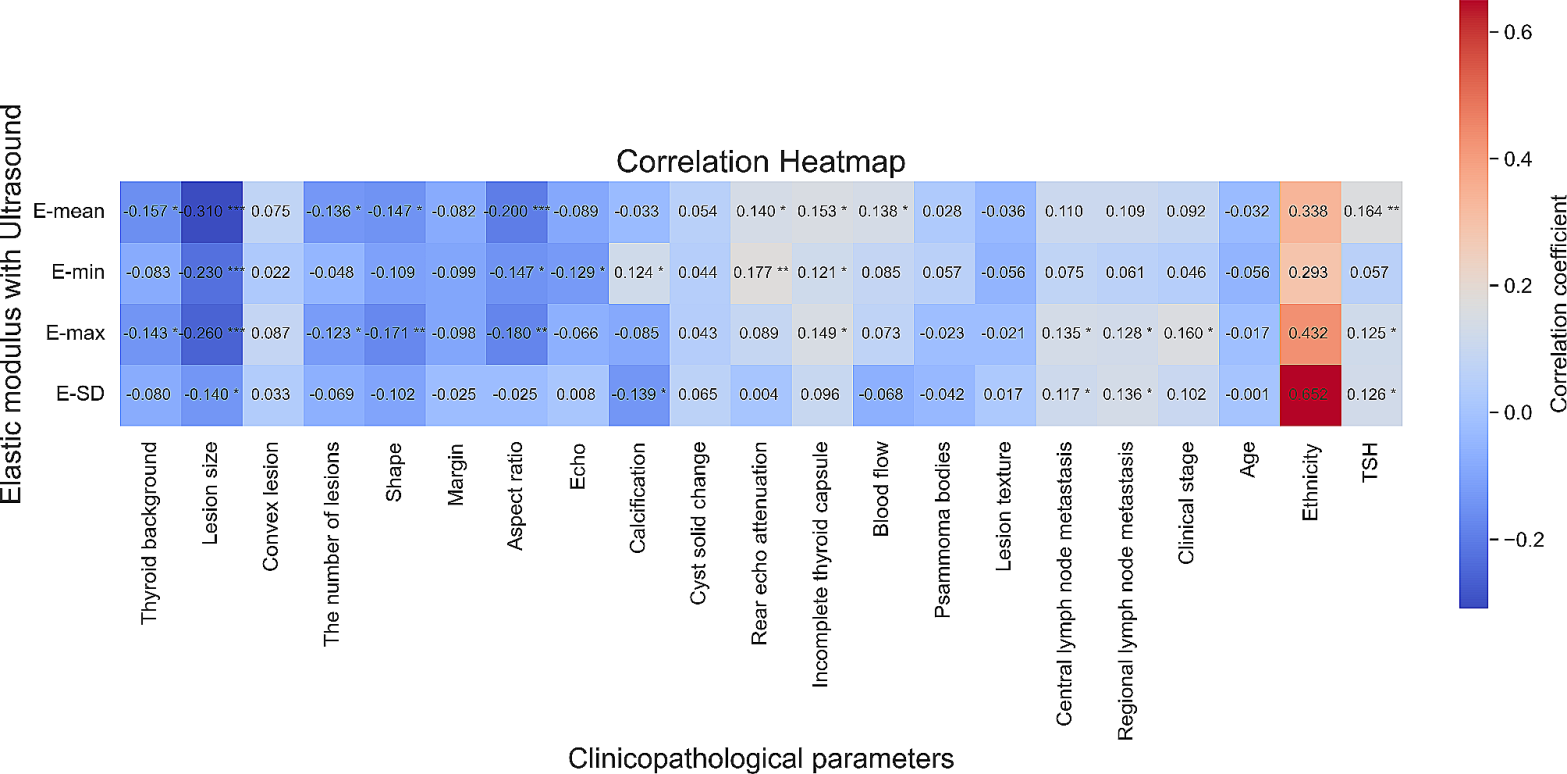
The heatmap illustrates the correlation between clinical pathological parameters and ultrasound elasticity modulus. Blue indicates negative correlation, red indicates positive correlation, and white indicates no correlation. The numerical values in the middle represent correlation coefficients. “*”denotes significance levels, where “*” indicates *P* < 0.05, “**” indicates *P* < 0.005, and “***” indicates *P* < 0.001. The factors of heterogeneous thyroid tissue background echo, lesions greater than 1 cm, multiple lesions, irregular shape, aspect ratio greater than 1, rear echo attenuation, incomplete thyroid capsule, intranodular blood flow within the lesions, central and regional cervical lymph node metastasis, advanced clinical stage more than stage 1, and increased TSH were high risk factors for the hardness of PTC lesions

According to previous study, the E-mean and E-max are most representative of the SWE elastic modulus [9]. The SWE image of PTC with high elastic modulus was shown in Fig. 2A and that with low elastic modulus was shown in Fig. 2B. In this study, the significant correlation of E-mean or E-max with ultrasonographic and clinicopathological features were analyzed and were defined as significant correlation. As shown in Table 3, the factors of heterogeneous thyroid tissue background echo, lesions greater than 1 cm, multiple lesions, irregular shape, aspect ratio greater than 1, rear echo attenuation, incomplete thyroid capsule, intranodular blood flow within the lesions, central and regional cervical lymph node metastasis, advanced clinical stage more than stage 1, and increased TSH were high risk factors for the hardness of PTC lesions. The E-SD value is an index to evaluate the internal homogeneity of lesions [17]. The larger the E-SD was, the more heterogeneous the lesions were. Our results showed that the larger lesions, calcification, and cervical lymph node metastasis were related with heterogeneous structure of thyroid cancer lesions(Table 3). The heatmap illustrating the correlation between clinical pathological parameters and ultrasound elasticity modulus is shown in Fig. 3.

**Table 3:** The correlation of PTC elastic modulus with ultrasound and clinicopathological parameters

Variables	E-mean		E-min		E-max		E-SD	
	*r*	*P*	*r*	*P*	*r*	*P*	*r*	*P*
Thyroid background	-0.157	0.006^**^	-0.083	0.250	-0.143	0.012^*^	-0.080	0.162
Lesion size	-0.310	< 0.001^**^	-0.230	< 0.001^**^	-0.260	< 0.001^**^	-0.140	0.014^*^
Convex lesion	0.075	0.191	0.022	0.697	0.087	0.130	0.033	0.565
The number of lesions	-0.136	0.017^*^	-0.048	0.406	-0.123	0.032^*^	-0.069	0.227
Shape	-0.147	0.010^*^	-0.109	0.057	-0.171	0.003^**^	-0.102	0.075
Margin	-0.082	0.154	-0.099	0.085	-0.098	0.088	-0.025	0.666
Aspect ratio	-0.200	< 0.001^**^	-0.147	0.010^*^	-0.180	0.002^**^	-0.025	0.661
Echo	-0.089	0.120	-0.129	0.025^*^	-0.066	0.252	0.008	0.885
Calcification	-0.033	0.563	0.124	0.030^*^	-0.085	0.139	-0.139	0.015^*^
Cyst solid change	0.054	0.352	0.044	0.448	0.043	0.454	0.065	0.255
Rear echo attenuation	0.140	0.015^*^	0.177	0.002^**^	0.089	0.120	0.004	0.940
Incomplete thyroid capsule	0.153	0.007^**^	0.121	0.034^*^	0.149	0.009^**^	0.096	0.093
Blood flow	0.138	0.016^*^	0.085	0.136	0.073	0.201	-0.068	0.237
Psammoma bodies	0.028	0.625	0.057	0.324	-0.023	0.684	-0.042	0.459
Lesion texture	-0.036	0.531	-0.056	0.326	-0.021	0.713	0.017	0.773
Central lymph node metastasis	0.110	0.054	0.075	0.192	0.135	0.018^*^	0.117	0.042^*^
Regional lymph node metastasis	0.109	0.057	0.061	0.289	0.128	0.025^*^	0.136	0.017^*^
Clinical stage	0.092	0.108	0.046	0.427	0.160	0.005^**^	0.102	0.076
Age	-0.032	0.583	-0.056	0.333	-0.017	0.762	-0.001	0.991
Ethnicity	0.338	0.713	0.293	0.276	0.432	0.650	0.652	0.522
TSH	0.164	0.004^**^	0.057	0.321	0.125	0.028^*^	0.126	0.028^*^

Note: *p < 0.05, **p < 0.01. TSH, thyroid stimulating hormone; E-mean/E-min/E-max/E-SD, the mean/minimum/max/ standard deviation value of SWE Young’s modulus value

### The relationship between PTC elastic modulus and the expression of CTGF

**Fig. 4:**
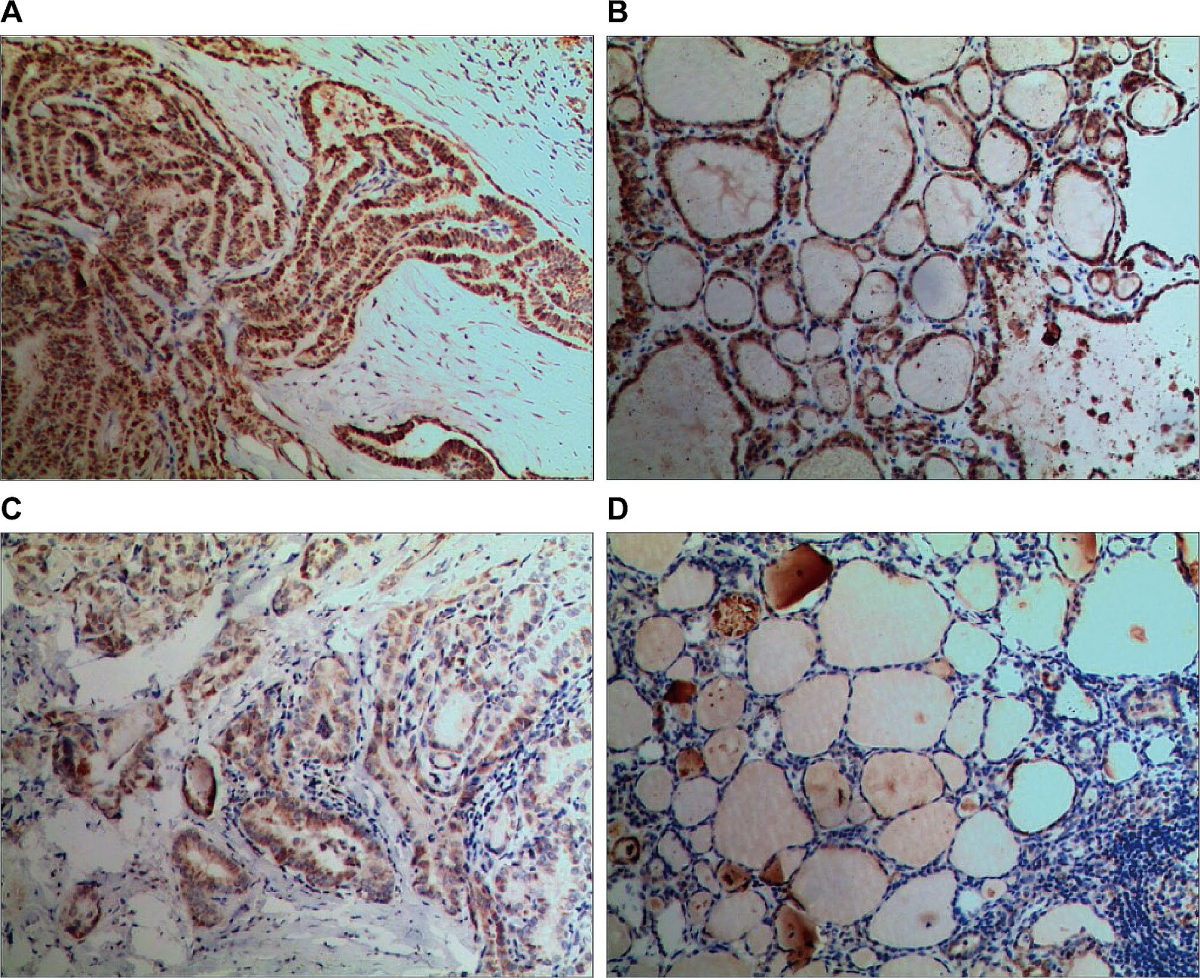
Analysis of CTGF expression. Immunohistochemistry was used to detect CTGF expression (×200). **(A)** High CTGF expression (+++) in PTC lesion with high elastic modulus. **(B)** Negative CTGF expression in tumor adjacent tissues of PTC lesion with high elastic modulus. **(C)** Weak positive CTGF expression (+) in PTC lesion with low elastic modulus. **(D)** Negative CTGF expression in tumor adjacent tissues of PTC lesion with low elastic modulus

The CTGF expression was detected by immunohistochemistry. The representative images were shown in Fig. 4. To evaluate the relationship between the fibrosis of PTC lesions and elastic modulus, we analyzed the expression levels of CTGF with their corresponding elastic modulus. We found that there were significant differences in the value of E-mean, E-min, E-max and E-SD of PTC lesions with various CTGF expression level (*P* < 0.01). The PTC lesions with high CTGF expression level had higher elastic modulus value than those with low CTGF expression (Table 4).

**Table 4:** Elastic modulus value of PTC with different CTGF expression

Elastic modulus value	Mean	CTGF				F	*P*
		-	+	++	+++		
E-mean	37.44 ± 22.59	25.94 ± 14.18	26.73 ± 11.55	33.35 ± 21.39	48.86 ± 24.01	22.114	< 0.001
E-min	20.00 ± 13.25	13.29 ± 12.17	15.17 ± 9.27	17.44 ± 11.08	26.29 ± 13.97	18.613	< 0.001
E-max	55.36 ± 39.52	41.70 ± 15.99	37.49 ± 15.25	50.55 ± 39.30	70.82 ± 46.15	12.253	< 0.001
E-SD	8.12 ± 8.58	6.61 ± 3.51	5.16 ± 2.92	7.55 ± 8.28	10.27 ± 10.82	4.827	0.003

Note: CTGF, connective tissue growth factor; E-mean/E-min/E-max/E-SD, the mean/minimum/max/ standard deviation value of SWE Young’s modulus value

### The relationship of CTGF with ultrasound and clinicopathological parameters

CTGF was expressed 85.95% of PTC patients (263/306). In order to clarify the relationship between the fibrosis level and ultrasonographic and clinicopathological parameters in PTC, we analyzed the expression of CTGF in tissue sections. In this study, increased expression of CTGF in PTC lesions was significantly associated with elevated TSH, convex and irregular shape, clear margin, aspect ratio larger than 1 and incomplete thyroid capsule (*P* < 0.05) (Table 5). The CTGF expression level had no association with the thyroid background, the lesion size, multiple lesions, echo, calcification, cystic solid change, rear echo attenuation, Intranodular blood flow, thyroid nodule status, psammoma bodies, lesion texture, lymph node metastasis, advanced clinical stage, age greater than 45 years old, gender and ethnic groups (*P* > 0.05) (Table 5).

**Table 5:** The expression of CTGF in PTC with different ultrasound and clinicopathological parameters

parameters	*n*	CTGF				χ^2^	*P*
		-	+	++	+++		
Thyroid background						4.346	0.226
Hashimoto’s thyroiditis	65	7	5	24	29		
Normal	241	36	39	75	91		
Lesion size						0.767	0.857
> 1 cm	109	16	16	32	45		
< 1 com	197	27	29	67	74		
Convex lesions						43.949	< 0.001
Nonconvex	255	40	43	93	79		
Convex	51	3	1	6	41		
The number of lesions						4.966	0.174
Multiple	79	9	8	33	29		
Single	227	34	36	66	91		
Lesion shape						23.496	< 0.001
Irregular	255	26	35	85	109		
Regular	51	17	10	14	10		
Margin						9.344	0.025
Blurred	266	32	38	86	110		
Clear	40	11	7	13	9		
Aspect ratio						7.972	0.047
< 1	54	4	4	17	29		
≥ 1	251	39	40	82	90		
Echo						0.989	0.804
Heterogeneity	291	41	44	93	113		
Homogeneity	15	2	1	6	6		
Calcification						2.405	0.493
No	54	7	6	15	26		
Yes	251	36	38	84	93		
Cystic solid change						0.952	0.813
No	301	42	44	97	118		
Yes	5	1	0	2	2		
Rear echo attenuation						1.923	0.589
No attenuation	51	6	10	14	21		
Attenuation	254	37	34	85	98		
Thyroid capsule						12.660	0.005
Incomplete	281	34	43	94	110		
Intact	25	9	1	5	10		
Blood flow						2.183	0.535
Perinodular blood flow	161	26	22	48	65		
Intranodular blood flow	145	17	23	51	54		
Thyroid nodule status						2.555	0.465
Hot	92	14	12	25	41		
Cold	214	29	33	74	78		
Psammoma bodies						4.533	0.21
No	177	25	22	53	77		
Yes	129	18	23	46	42		
Lesion texture						9.142	0.16
Soft	30	5	7	6	12		
Hard	130	24	19	38	49		
Tough	146	14	19	55	58		
Central lymph node Metastasis						4.997	0.172
No metastasis	157	21	27	43	66		
Metastasis	149	22	17	56	54		
Regional lymph node metastasis						9.181	0.164
No metastasis	166	26	27	46	67		
Central metastasis	94	9	15	32	38		
Central and cervical metastasis	46	8	3	21	14		
Clinical stage						0.975	0.807
Stage 1	227	31	36	73	87		
Stage 2–4	79	12	9	26	32		
Age						3.346	0.341
< 45	152	20	23	43	66		
> 45	154	23	22	56	53		
Gender						7.062	0.070
Male	78	14	8	31	25		
Female	228	29	37	68	94		
Ethnicity						8.159	0.227
Han	224	25	36	72	91		
Uighur	47	11	6	13	17		
Others	35	7	3	14	11		
TSH						28.087	< 0.001
Reduced	23	8	6	3	6		
Normal	202	28	29	78	67		
Increased	81	7	10	18	46		

Note: CTGF, connective tissue growth factor; TSH, thyroid stimulating hormone

### Multivariate correlation analysis of CTGF, SWE modulus, conventional ultrasound and clinicopathological features

**Fig. 5:**
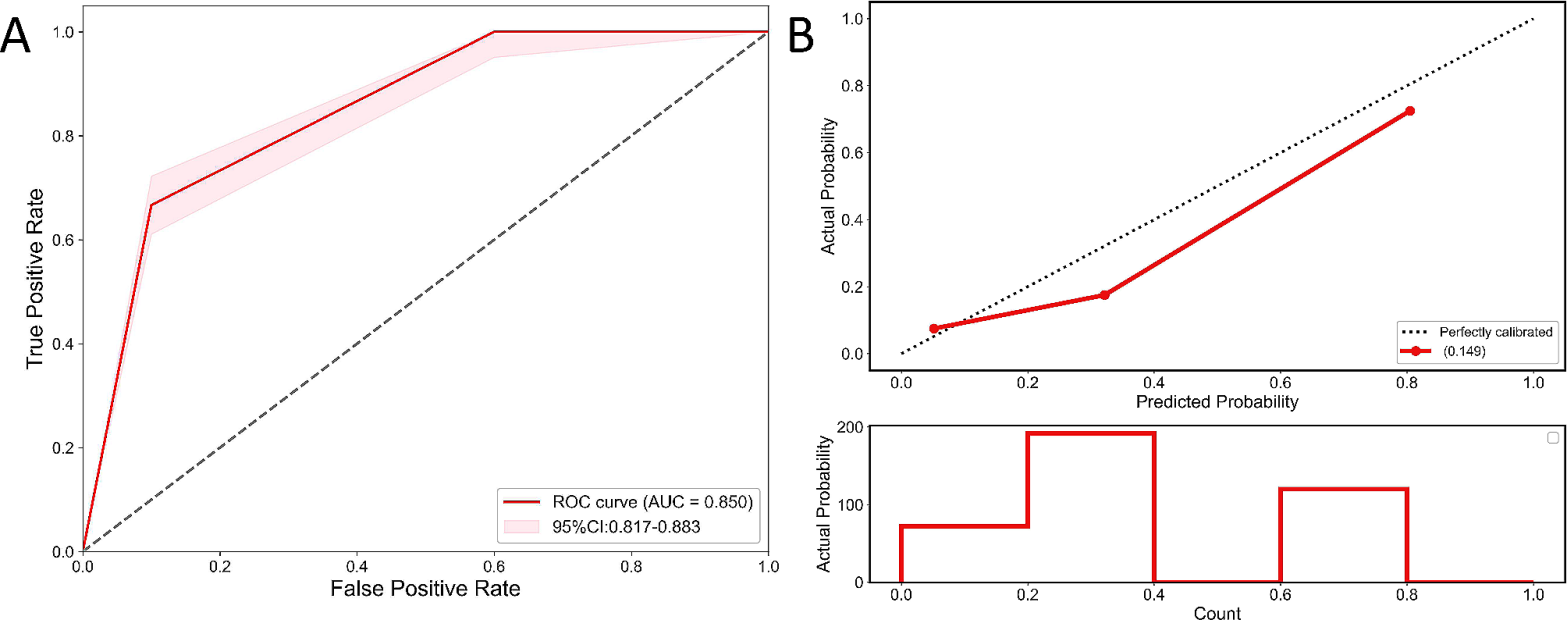
ROC and calibration curves of the regression model. **(A)** ROC curve of the regression model, with an AUC of 0.850 and a 95% CI of 0.817–0.883. **(B)** Calibration curve of the regression model, where the X-axis represents predicted probability and the Y-axis represents observed probability. The 45-degree “ideal” line represents perfect prediction for CTFG, while the “bias correction” line represents the regression model. The closer the “bias correction” line is to the “ideal” line, the better the performance

In order to get rid of the confounding factors, we conducted a multivariate analysis. The dependent variable of this regression was the expression level of CTGF, while the independent variables were the factors that were statistically significant in the univariate analysis and correlation analysis. The results demonstrate that the Area Under Curve(AUC) value of the logistic regression is 0.850 (95% CI: 0.817, 0.883), with sensitivity of 0.667 (95% CI: 0.590, 0.744) and specificity of 0.900 (95% CI: 0.862, 0.938). The optimal cutoff value is 0.461. The ROC curve and calibration curve are depicted in Fig. 5A and B, respectively. The logistic regression model found that irregular shape (OR = 2.871, CI: 1.149–7.177, *P* < 0.05), aspect ratio ≥ 1 (OR = 2.524, CI: 1.300-4.901, *P* < 0.05) and elevated TSH (OR = 0.304, CI: 0.173–0.536, *P* < 0.05) were significantly different (Table 6). The risk factors were included into the equation according to their contribution order. The equation was Logit (P) = 1.153 + 1.055 × 1 + 0.926 × 2 + 1.190 × 3 (X1: irregular shape; X2: aspect ratio ≥ 1; X3: elevated TSH). Likelihood ratio test showed that the model was statistically significant (χ2 = 35.290, *P* < 0.001) and R^2^ = 0.675. Although factors including convex lesions, blurred margin and incomplete thyroid capsule were significant in univariate analysis, the regression model indicated that they were not independent risk factors of the high expression of CTGF.

**Table 6:** Multivariable correlation analysis of CTGF, SWE modulus, conventional ultrasound and clinicopathological parameters

Parameters	B	S.E,	Wals	df	Sig.	OR	95% C.I.	
							Lower limit	Upper limit
Aspect ratio	0.926	0.339	7.483	1	0.006	2.524	1.300	4.901
Shape	1.055	0.467	5.091	1	0.024	2.871	1.149	7.177
Margin	0.402	0.503	0.638	1	0.425	1.495	0.557	4.009
Capsule invasion	0.340	0.571	0.354	1	0.552	1.405	0.459	4.304
TSH			17.766	2	0.000			
Reduced TSH *Vs.* normal TSH	-1.246	0.542	5.285	1	0.052	0.288	0.099	0.832
Increased TSH *Vs.* normal TSH	1.190	0.289	16.965	1	0.000	0.304	0.173	0.536
Convex lesions	0.186	0.670	0.077	1	0.781	0.830	0.223	3.087
constant	1.153	0.757	2.316	1	0.128	0.316		

Note: TSH, thyroid stimulating hormone;

## Discussion

### The value of SWE combined with CTGF in the risk assessment of PTC

The results of this study showed that male, Uygur ethnicity, convex lesions, calcification, incomplete capsule, intranodular blood flow, rear echo attenuation, cervical lymph node metastasis, lesions greater than 1 cm, psammoma bodies, advanced clinical stage, and, increased TSH indicated poor prognosis. This result is consistent with previous study [18], except for ethnicity. Previous report found that gender was also an independent risk factor for the incidence of thyroid cancer [19] and in this study we found that the ethnic groups also played the role. Besides, other risk factors of PTC included tumor size, calcification, and blood flow distribution. The tumor size reflects tumor growth activity and larger tumors have a tendency to develop aggressive biological behavior [20]. Micro-calcification within tumor lesions called psammoma body is an important pathological characteristic of PTC, which are deposited calcium salt crystals of 10–100 μm in size [21]. Thyroid nodules supplied with more central blood flow signals imply malignant tissues with good specificity but unsatisfactory sensitivity [22]. Another important finding from our study was that both SWE and CTGF were valuable factors to assess the risk of PTC. The Young’s modulus value of the tissue is calculated according to the propagation velocity of the shear wave in different tissues, which reflects the hardness of the tumor [23]. In addition, the expression of CTGF is closely related to the incidence, progression, metastasis, invasion, tumor angiogenesis and prognosis of thyroid cancer [24]. Both CTGF and SWE are closely related to the fibrosis level of thyroid cancer.

### The relationship of the hardness of PTC lesions and ultrasound and clinicopathological indicators

The E-mean could fully reflect the hardness of thyroid nodules, but factors such as nodular coarse calcification and liquefaction may affect E-mean [25]. The E-max only reflects the hardness of the hardest area of the lesion but not the hardness of the lesion itself [25]. In this study, we found that the calcification of PTC lesions was related to the minimum and standard deviation of Young’s modulus but not related to the mean and maximum values. It may be because the cases of gross calcification were not excluded in this study.

This study showed that the risk factors of PTC lesion hardness were inconsistent with the prognostic factors of PTC. Both thyroid background and the number of lesions were risk factors of PTC lesions but other factors including gender, ethnicity, calcification and psammoma bodies were not. Therefore, the factors related to the hardness of PTC lesions included the main indicators of ultrasound in the diagnosis of benign or malignant tumor. It is worth noting that the hardness of PTC was related with Hashimoto’s thyroiditis and elevated TSH. In the progress of Hashimoto’s thyroiditis, the infiltration of lymphocytes and plasma cells is large, leading to follicular destruction and tissue fibrosis. As a result, the rigidity of glandular tissue increases together with more heterogeneous background of PTC lesion and elevated elasticity modulus. On the other hand, TSH reflects the progress of Hashimoto’s thyroiditis, which is consistent with the fibrosis level of Hashimoto’s thyroiditis. Meanwhile, elevated TSH also has significant tumorigenic effect [26].

### CTGF correlation with ultrasound index

This study detected that there was a significant correlation between the expression of CTGF and the main conventional malignant features of ultrasound in PTC, indicating that the main indicators of benign and malignant thyroid nodules by conventional ultrasound can predict the prognosis of thyroid nodules. Echogenic structures within the PTC lesion include echo-homogeneity, calcification, liquefaction, cystic change, rear echo attenuation, and intranodular blood flow. It appears as hypoecho if the fibrosis of the nodules is increased accompanied by the reduction of the follicles [27]. As a result, PTC shows low echo and rear echo attenuation due to approximately 90% of thyroid papillary carcinoma with obvious fibrous tissue hyperplasia [28]. Another influencing factor of the echoes of PTC lesions is the arrangement of fibrous tissue growth [28]. Based on the univariate analysis in the present study, the irregular morphology, unclear margin, aspect ratio ≥ 1, convex lesions and incomplete thyroid capsule tended to have higher CTGF expression and higher fibrosis degree. The expression of CTGF is unrelated to the hot and cold nodules of PTC. This may be due to CTGF exerting its effects through various mechanisms such as promoting cell proliferation and influencing the extracellular matrix [24]. Most PTCs present as cold nodules, characterized by low enhancement on ultrasound contrast imaging, which is attributed to differences in microvascular density compared to surrounding tissues [29]. Therefore, in this study, there was no significant difference in CTGF expression between hot and cold nodules of PTC. It is worth noting that there is limited research data on CTGF in PTC, and further experimental validation with larger sample sizes is needed to elucidate its expression and its association with hot and cold nodules of PTC. CTGF expression was not significantly related with the hardness of PTC lesions, however, it was significantly related with E-mean, E-min, E-max and E-SD. This result indicates that the hardness of PTC lesions is subjective, and the hardness measured by SWE is more objective and can quantify the hardness. CTGF was not related with thyroid background (hashimoto’s thyroiditis / normal). However, the hardness of PTC lesion was associated with CTGF thyroid background (hashimoto’s thyroiditis / normal). This may be related to the fact that the PTC lesion contains part of the tissues of hashimoto’s thyroiditis. CTGF was not related to calcification and psammoma bodies, but it was related to lesion hardness. It may be due to the inclusion of calcification in the PTC lesion and the exclusion of CTFG in CTGF.

CTGF is reported form a complex with VEGF165 and inhibits VEGF-induced angiogenesis [30], leading to inhibition of tumor angiogenesis and metastasis. However, this study suggested that CTGF expression levels exhibited statistically insignificant relations with blood supply of the PTC in ultrasound. It may be due to vast majority of thin blood vessels in PTC. Previous study [31] reports that PTC vessels are characterized with lack of blood supply resulted from the vessels with small diameters (approximately 2–3 μm).

### CTGF correlation with clinical parameters

Elevated TSH level may be a potential risk factor of thyroid carcinoma. TSH is an important hormone of thyroid function, which can promote thyroid follicular epithelial cell proliferation and thyroid hormone synthesis and release. Excessive TSH secretion can increase the risk of tumorigenesis [32]. This study showed that there was a significant correlation between CTGF expression and TSH levels in patients with PTC. PTC lesions with elevated TSH expressed high levels of CTGF, suggesting that high expression of CTGF not only had a poor prognosis, might also be associated with hypothyroidism. The reasons may be as follows: PTC may be accompanied by thyroid inflammation and fibrosis, which may influence the expression of CTGF through the interaction of cytokines and growth factors in the microenvironment [33]. Additionally, these changes may also affect the secretion and regulation of TSH. TSH, as the main hormone regulating thyroid function, is influenced by the thyroid functional status [34]. Furthermore, as a factor promoting fibrosis, overexpression of CTGF may exacerbate inflammation and fibrosis in thyroid tissue, leading to elevated TSH levels.

In this study, CTGF was insignificantly related to age, gender, ethnicity, lymph nodes or clinical stage, which suggests that the degree of fibrosis is related with the pathological structure of the lesions, but not with the individual characteristics. In the multivariate analysis of CTGF, only the irregular margin, the aspect ratio ≥ 1 and elevated TSH were independent factors. Other ultrasound signs including convex shape, unclear boundaries and incomplete thyroid capsule had no significance, which indicates that they are subjective and confounding variables.

## Conclusions

In conclusion, SWE and CTGF were of great value in the assessment of PTC risk. The degree of fibrosis in PTC was closely related to the prognosis. The hardness of PTC lesions and the expression level of CTGF were all correlated with the main indexes of differentiating benign or malignant thyroid lesions in conventional ultrasound, indicating that ultrasound signs could predict the prognosis. PTC lesion hardness had many influencing factors. Irregular shape, aspect ratio ≥ 1 and increased TSH level were independent factors of CTGF. The degree of fibrosis of the lesion was related to the pathological structure of the lesion itself, but not to the individual characteristics. High expression of CTGF was not only a poor prognosis of PTC but also be associated with hypothyroidism.

## Data Availability

The datasets used and/or analysed during the current study are available from the corresponding author on reasonable request.

## Abbreviations

SWE: Shear wave elastography
CTGF: Connective tissue growth factor
PTC: Papillary thyroid carcinoma
TSH: Thyroid stimulating hormone
E-max: The max value of shear wave speed imagine
E-min: The minimum value of shear wave speed imagine
E-mean: The mean value of shear wave speed imagine
E-SD: The standard deviation of modulus of shear wave speed imagine
AUC: Area under the curve
ROC: Receiver operating characteristic
